# Interleukin-12 (IL-12p70) Promotes Induction of Highly Potent Th1-Like CD4^+^CD25^+^ T Regulatory Cells That Inhibit Allograft Rejection in Unmodified Recipients

**DOI:** 10.3389/fimmu.2014.00190

**Published:** 2014-05-09

**Authors:** Nirupama Darshan Verma, Bruce Milne Hall, Karren Michelle Plain, Catherine M. Robinson, Rochelle Boyd, Giang T. Tran, Chuanmin Wang, G. Alex Bishop, Suzanne J. Hodgkinson

**Affiliations:** ^1^Immune Tolerance Laboratory, Department of Medicine, Liverpool Hospital, University of New South Wales, Kensington, NSW, Australia; ^2^Collaborative Transplant Research Laboratory, Royal Prince Alfred Hospital, The University of Sydney, Camperdown, NSW, Australia

**Keywords:** T regulatory cells, antigen-specific Treg, Th1-like Treg, allograft rejection, IL-12p70

## Abstract

In rat models, CD4^+^CD25^+^ T regulatory cells (Treg) play a key role in the induction and maintenance of antigen-specific transplant tolerance, especially in DA rats with PVG cardiac allografts (1, 2). We have previously described generation of alloantigen-specific Treg (Ts1), by culture of naïve natural CD4^+^CD25^+^ Treg (nTreg) with specific alloantigen and IL-2 for 4 days. These cells express mRNA for IFN-γ receptor (*ifngr*) and suppress donor but not third party cardiac allograft rejection mediated by alloreactive CD4^+^ T cells at ratios of <1:10. Here, we show that Ts1 also expressed the IL-12p70 specific receptor (il-12rβ2) and that rIL-12p70 can induce their proliferation. Ts1 cells re-cultured with rIL-12p70 alone or rIL-12p70 and recombinant interleukin-2 (rIL-2), suppressed proliferation of CD4^+^ T cells in mixed lymphocyte culture at <1:1024, whereas Ts1 cells re-cultured with rIL-2 and alloantigen only suppressed at 1:32–64. The rIL-12p70 alloactivated Ts1 cells markedly delayed PVG, but not third party Lewis, cardiac allograft rejection in normal DA recipients. Ts1 cells re-cultured for 4 days with rIL-12p70 alone, but not those re-cultured with rIL-12p70 and rIL-2, expressed more *il-12rβ2, t-bet*, and *ifn-γ*, and continued to express the markers of Ts1 cells, *foxp3, ifngr*, and *il-5* indicating Th1-like Treg were induced. Ts1 cells re-cultured with rIL-2 and alloantigen remained of the Ts1 phenotype and did not suppress cardiac graft rejection in normal DA rats. We induced highly suppressive Th1-like Treg from naïve nTreg in 7 days by culture with alloantigen, first with rIL-2 then with rIL-12p70. These Th1-like Treg delayed specific donor allograft rejection demonstrating therapeutic potential.

## Introduction

Antigen-specific CD4^+^CD25^+^ T regulatory cells (Treg) mediate transplant tolerance (3–5) and can protect against autoimmunity (6). Natural CD4^+^CD25^+^ Treg (nTreg) prevent autoimmunity (7) and contribute to the induction of transplant tolerance (8). However, nTreg mediated suppression is not antigen-specific (9, 10) and requires high ratios, usually >1:1 of nTreg:effector CD4^+^ T cells to inhibit organ allograft rejection (2, 8) or graft versus host disease (GVHD) (11). This required ratio is much higher than the 1:10 or less that is in peripheral lymphoid tissues of normal and transplant tolerant animals (4). There is tight homeostatic control of the ratio of CD4^+^CD25^+^ T cells to CD4^+^CD25^−^ T cells in peripheral lymphoid tissues, thus alloantigen-specific CD4^+^CD25^+^ Treg maintain tolerance to the allograft at ratios of <1:10 (4). Thus antigen-specific Treg suppress at ratios of <1:10 and would need fewer cells to induce tolerance than with nTreg.

nTreg *in vitro* can be massively expanded using current culture techniques with IL-2 and stimulation with anti-CD3 alone or combined with anti-CD28 monoclonal antibodies (mAb) (12), but these Treg only suppress at ratios of >1:1. Thus, extremely large numbers of nTreg are required *in vivo* to prevent allograft rejection and GVHD in unmodified recipients (13). The number of expanded nTreg required is so high it may be impossible to achieve in humans (13). Methods that induce more potent antigen-specific Treg, that can suppress at lower ratios and thus require fewer Treg would be desirable.

We previously reported that nTreg cultured with recombinant interleukin-2 (rIL-2) and alloantigen produce alloantigen-specific Treg that suppress *in vitro* and *in vivo* (2, 14–16). These antigen-specific Treg, suppress *in vivo* at lower ratios of 1:10.

Our earlier studies show CD4^+^ T cells that transfer transplant tolerance are short lived *in vitro* (3, 17, 18) but their suppressive potential is preserved by culture with donor specific alloantigen and lymphocyte derived cytokines (18). The specific cytokines required are not completely characterized, but IL-2 (18) or IL-4 (19) alone are not sufficient to fully maintain CD4^+^ Treg that transfer transplant tolerance (18). This suggests that induction and expansion of antigen-specific Treg, that can maintain transplant tolerance, may depend on cytokines other than IL-2 or IL-4 long term, albeit there initial activation requires either IL-2 or IL-4.

We have previously reported that short term cultures of nTreg with either rIL-2 or rIL-4 and alloantigen for 3–4 days induce alloantigen-specific Treg that inhibit specific donor but not third party fully allogeneic heart graft rejection at ratios of 1:10 (2). They also inhibit proliferation of CD4^+^ T cells to specific donor more than to third party in mixed lymphocyte culture (MLC) (2). Culture of nTreg with rIL-2 and alloantigen induced expression of IFN-γ receptor (ifngr) and il-5, but reduced ifn-γ expression (2). As the abbreviation Tr1 had been designated to IL-10 activated Treg, we named these rIL-2 activated Treg as Ts1 that express receptors for Th1 cytokines (2). Activation of nTreg with rIL-4 and alloantigen generated activated Tregs expressing il-5rα, a Th2 cytokine receptor; not ifngr, and were named Ts2 (2).

The pathways by which nTreg may be activated by Th1 or Th2 cytokines to produce antigen-specific Treg, has recently been reviewed (1). There is increasing evidence that Treg function could be influenced by Th1 cytokines, other that IL-2. Activated Treg express ifngr (20) and the receptor for IL-12p70 (il-12rβ2) (21). IL-12Rβ2^−/−^ mice develop more rapid and severe autoimmune disease than wild-type (22) due to reduced CD4^+^CD25^+^ Treg activity (21). In uncontrolled Th1 responses, IFN-γ and IL-12p70 induce Treg to express t-bet and ifn-γ while they continue to express foxp3 and suppress (23, 24). These rIL-12p70 activated Treg are called Th1-like Treg, because they express the Th1 transcription factor *t-bet* and the Th1 cytokine *ifn-γ* as well as *foxp3* but do not produce IL-2. Induction of Th1-like Treg by IL-12p70 does not occur in the presence of IL-2 (21, 25). Th1-like Treg have been described in patients with multiple sclerosis (26) and renal transplants (27). The precise role of Th1-like Treg is not understood.

Here we described that Ts1 cells also expressed mRNA for *il-12rβ2*, the specific receptor for IL-12p70, and their culture with rIL-12p70 and alloantigen resulted in their proliferation and further activation into Th1-like Treg, expressing *t-bet, ifn-γ* and *foxp3, ifngr* and *il-12rβ2*. These Th1-like Treg were much more potent than nTreg or Ts1 in suppressing CD4^+^ T cell responses to alloantigen *in vitro* and *in vivo*. We have selectively expanded alloantigen-specific Treg, a situation that will have only a minority of cells growing, therefore there were lower rates of proliferation than with polyclonal expansion of nTreg.

## Materials and Methods

### Animals

DA (RT1a), PVG (RT1c), and Lewis (RT-1l) rats were bred and maintained in the animal house, Liverpool Hospital. Heterotopic heart grafts were performed as described (28). Experiments were approved by the Animal Ethics Committee of the University of New South Wales.

### Monoclonal antibodies and cytokines

Anti-rat mAb used were W3/25 (CD4), MRCOx8 (CD8), MRCOx39 (CD25), MRCOx33 (CD45RA), MRCOx6 (class II MHC) (BD-PharMingen, San Diego, CA, USA), and FITC anti-mouse/rat Foxp3 (eBioscience, San Diego, CA, USA). Subsets of T cells were identified by indirect immunofluorescence staining and enumerated by gating the whole lymphocyte population on a FACScan, as described (9).

Rat recombinant, rIL-12p70, rIL-12p40, and rIL-2 were prepared and assayed as described (29). Briefly, rat IL-12p35 and IL-12p40 were cloned from rat spleen cells and transfected alone or together into Chinese Hamster Ovary cells (CHOK1). rIL-12p70 was produced from CHOK1 cells lines transfected with both p35 and p40, as described (29). Cytokines were produced from stably transfected CHOK1 cells grown to confluence in DMEM-F12 medium (GIBCO, Life Technology, Grand Island, NY, USA) with 10% FCS (Trace Biosciences, Castle Hill, NSW, Australia), then washed and re-cultured in serum free medium. After 4–5 days, the medium was harvested and assayed for capacity to promote proliferation of IL-2/ConA activated rat spleen cells in culture for 4 days. One unit was defined as the cytokine that induced 50% maximum proliferation (29). rIL-4 transfected line (30) was a kind gift of Dr. Neil Barclay (School of Pathology, Oxford, UK) and was produced as supernatant from transfected CHOK1 cells lines grown in serum free medium and assessed in a bioassay for it ability to induce class II MHC on B cells, as described (2, 29).

### Preparation of T cell subsets

Lymph nodes and spleen cells from naïve DA rats were depleted of CD8^+^ T and B cells using mAb MRCOx8 (CD8) and MRCOx33 (CD45RA) and an indirect panning technique, as described (31). The CD4^+^CD25^+^ Treg population was prepared using PE conjugated MRCOx39 and mouse anti-PE microbeads (Miltenyi, Bergisch Gladbach, Germany) before eluting through a LS MACS column (Miltenyi), as described (2, 8, 9). The cells were 98–99% CD4^+^, 85–95% CD25^+^, and approximately 80% Foxp3^+^.

### Culture of CD4^+^CD25^+^ T cells to induce alloantigen-specific Treg (Ts1)

Cell culture medium used was RPMI 1640 (GIBCO) supplemented with 100 ng/ml penicillin, 100 U/ml streptomycin (Glaxo, Boronia, VIC, Australia), 2 mM l-glutamine, 5 × 10^−^5 M 2-mercaptoethanol (Sigma), and 20% Lewis rat serum. Stimulator cells were thymus cells of DA, PVG, or third party Lewis rats irradiated *in vitro* with 9 Gy, as described (32). Stimulator cells had <1% lymphocytes and background levels of mRNA for T cell cytokine (32). MLCs with naïve DA CD4^+^, CD4^+^CD25^−^, or CD4^+^CD25^+^ T cells were performed, as described (9), that had either no cytokine, rIL-2 (200 units/ml) or rIL-4 (200 units/ml) alone or with rIL-12p70 (20 units/ml). 200 units/ml of rIL-2 or rIL-4 is the optimal concentration for activation of nTreg (2). Cultures in U-bottom microtiter plates (Greiner, Frickenhausen, Germany) had 2 × 10^4^ stimulator cells and 10^5^ responder T cells per well. Six replicate wells were set up for each group.

For bulk cultures, naïve DA CD4^+^CD25^+^ Treg (2 × 10^6^/ml) were cultured with irradiated thymus stimulator cells (10^6^/ml) from PVG rats in 25 cm^2^ flasks (Greiner). These when cultured for 3–4 days with rIL-2 or rIL-4 produced Ts1 or Ts2 cells, as described (2, 9). Ts1 cells were washed and further cultured with new PVG stimulator cells and rIL-2 alone (200 units/ml), rIL-12p70 alone (20 units/ml), rIL-2 and rIL-12p70 or rIL-12p40 for 3–4 days. After 3–4 days of culture, cells were assayed by FACS for cell surface markers, by RT-PCR for cytokine and cytokine receptor expression and by ^3^H-thymidine incorporation for cell proliferation assay and for their ability to inhibit naïve syngeneic CD4^+^ T or CD4^+^CD25^−^ T cell proliferation in MLC, as described (2).

The effects of blocking IFN-γ with the mAb DB-1 (33) (Ucytec, Utrecht, The Netherlands) at 50 μg/ml or iNOS with L-NIL (Sigma Chemicals, St. Louis, MO, USA) at 0.1 mg/ml on activation of Ts1 cells by rIL-12p70 was examined. DB-1 and L-NIL, at these concentrations have no effect on nTreg proliferation in MLC but enhance proliferation of CD4^+^CD25^−^ cells showing they block iNOS and NO production that inhibits cell proliferation (9).

### RT-PCR

RNA extraction, cDNA synthesis, and semi-quantitative PCR on a Rotorgene PCR machine (Corbett Research, Mortlake, NSW, Australia) were performed as described (2, 32). Known primers for rat *gapdh, il-2, ifn-γ, il-4, il-5* (32, 34), *ifngr, il-5rα, foxp3, t-bet, gata-3* (2), *il-12rβ2* (29), and SYBR Green I and HotMaster Taq polymerase (Eppendorf AG, Hamburg, Germany) or SensiMix DNA kit (Bioline, Alexandria, NSW, Australia) were used as described (32, 34). Gene copy number was derived from a standard curve run in parallel and normalized against *gapdh* as described (2).

### Suppressor assay

Microcultures in *U*-bottom micro-titer plates (Linbro, Flow Labs, VA, USA) were prepared with serial dilutions of Treg. To each well 2 × 10^4^ irradiated thymus stimulator cells either from syngeneic DA, specific donor PVG or third party Lewis and 10^5^ DA responder naïve CD4^+^ T cells or naïve CD4^+^CD25^−^ T cells, were added in a final total volume of 200 μl as previously described (9) (four to six replicate wells/treatment). Proliferation was measured by ^3^H-thymidine incorporation at day 4 as described (9). In rat MLC, CD4^+^ T cell proliferation is usually 3–10 × 10^3^ cpm, and autologous responses have low backgrounds of 2–3 × 10^3^ cpm, whereas CD4^+^CD25^−^ T cells have higher responses to self and alloantigen, as no nTreg cells are present (9).

### Cardiac allografts

Heterotopic PVG or Lewis hearts were grafted into naïve DA rats as described (28). Graft function was monitored by palpation, as described (2). In DA rats, prolonged graft survival is easier to induce with PVG (35) or Lewis (36) organ allografts than with other strains (37). Thus, Lewis grafts are an appropriate third party control. On day 3 post-transplant, recipient rats received 5 × 10^6^ Treg that had been activated with PVG stimulators and rIL-2 for 3 days followed by 4 days with PVG stimulators and either rIL-12p70 or rIL-2. The Treg were given at 3 days post-transplant as this allows the host response to the allograft to be activated, and the response to produce cytokines such as IL-12 that may be required to promote further expansion and survival of the Treg *in vivo*.

### Statistical analyses

Parametric data was expressed as mean ± standard deviation and assessed with a Student’s *t*-test, while non-parametric data was assessed with a Wilcoxon Rank Sum test on Statview (Abacus Concepts, Berkeley, CA, USA). Statistical significance was *p* < 0.05.

## Results

### Effect of rIL-12 p70 on proliferation of CD4^+^CD25^+^ T cells

**Figure 1:**
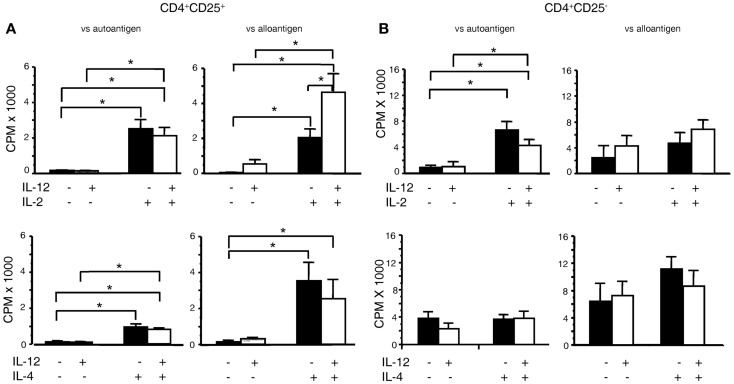
**Effect of rIL-12p70 on proliferation of CD4^+^CD25^+^ and CD4^+^CD25^−^ T cells in MLC supplemented with either rIL-2 or rIL-4**. **(A)** Effect of rIL-12p70, alone or in combination with either rIL-2 or rIL-4, on proliferation of **(A)** CD4^+^CD25^+^ T cells and **(B)** CD4^+^CD25^−^ T cells from naïve DA rats stimulated in culture with either self (DA) or allogeneic (PVG) stimulator cells. These were primary mixed lymphocyte cultures with proliferation assessed at day 4. The cells were fresh and had not been cultured before. Addition of rIL-12p70 enhanced proliferation of CD4^+^CD25^+^ T cells to alloantigen, not auto-antigen, in cultures with rIL-2 but not with rIL-4. rIL-12p70 had no significant effect on proliferation of CD4^+^CD25^−^ T cells, even when rIL-2 was present. *Significant differences (*p* < 0.05). Similar results have been obtained in other experiments.

The bioassay of IL-12p70 tests its ability to promote proliferation of IL-2 activated T cells (38). As nTreg proliferate with IL-2, we examined the effects of rIL-12p70 on proliferation of CD4^+^CD25^+^ Treg from DA rats stimulated with PVG alloantigens in presence of rIL-2 or rIL-4 (Figure 1A). Addition of either rIL-2 or rIL-4, enhanced proliferation of nTreg, as described (6). rIL-12p70 (20 units/ml) alone had no effect on proliferation but when added with rIL-2 enhanced proliferation compared to rIL-2 alone (*p* < 0.05). Combination of rIL-4 and rIL-12p70 did not enhance proliferation of nTreg, compared to rIL-4 alone. With autologous DA stimulator cells, rIL-2 and to a lesser extent rIL-4 induced proliferation of nTreg, but rIL-12p70 had no synergistic effect with either.

In parallel, we examined the effects of rIL-2 or rIL-4 alone or with rIL-12p70 on naïve CD4^+^CD25^−^ T cell proliferation to either auto- or alloantigen (Figure 1B). For allogeneic stimulators, rIL-12p70 combined with rIL-2 had a trend to increased proliferation compared to rIL-2 alone, and had no effect when combined with rIL-4. Naïve CD4^+^CD25^−^ T cells’ response to auto-antigen was enhanced by rIL-2, but not with rIL-4 or rIL-12p70.

### Expression of *il-12rβ2* by nTreg activated by rIL-2 (Ts1) but not rIL-4 (Ts2)

**Figure 2:**
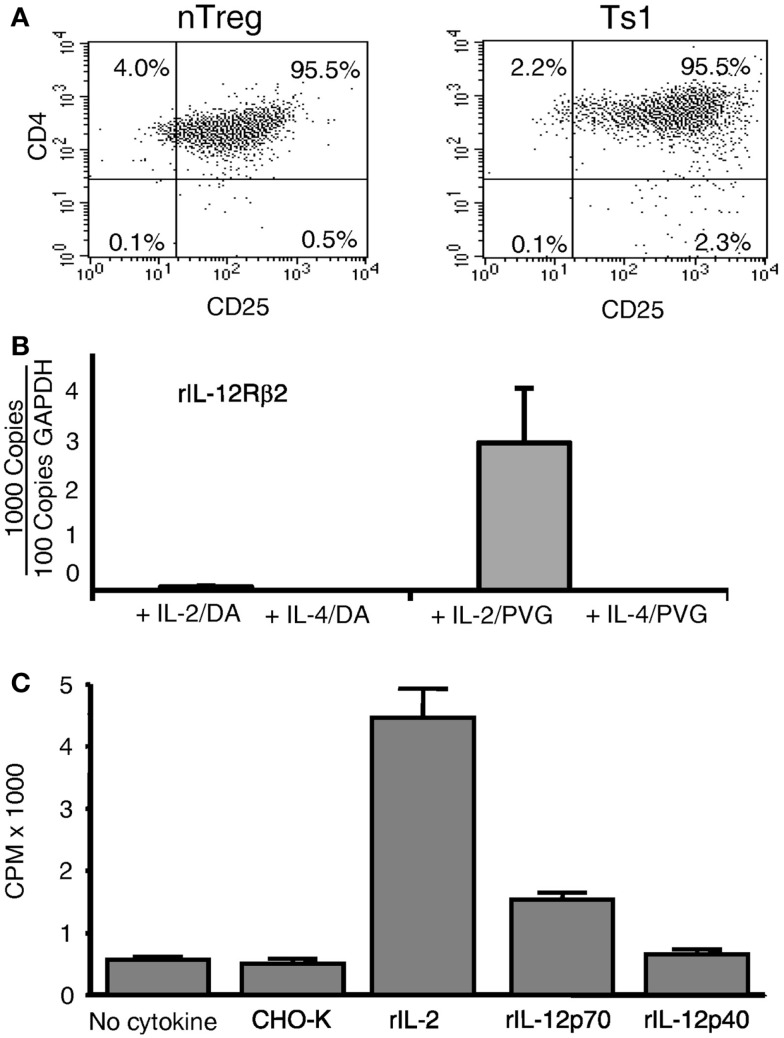
**Expression of CD25 and *il-12rβ2* and *in vitro* proliferation in MLC of naïve CD4^+^CD25^+^ T cells**. **(A)** Comparison of naïve CD4^+^CD25^+^ T cells before and after culture with rIL-2 and alloantigen. The majority of CD4^+^ T cells expressed CD25 before culture (nTreg) and remained CD25^+^ post-culture with rIL-2 and alloantigen (Ts1). Replicated in several experiments. **(B)** Expression of *il-12rβ2* in CD4^+^CD25^+^ T cells (nTreg) from DA rats cultured for 3 days with DA (auto) or PVG (allo) stimulators in the presence of rIL-2, rIL-4, or no cytokines. The CD25^+^ population was re-enriched to remove stimulator cells. *il-12rβ2* was increased in CD4^+^CD25^+^ T cells cultured with rIL-2 and alloantigen to induce Ts1 cells and to a lesser extent with autoantigen. There was no *il-12rβ2* expression in nTreg cultured with alloantigen alone with no cytokine as well as those cultured with rIL-4 and auto- or alloantigen. There was no expression of *il-2*, confirming no Th1 induction after culture with rIL-2, *ifngr* was induced, but not with rIL-4, as described (8) (data not shown). One of three experiments with similar results. **(C)** Proliferation of Ts1 cells re-cultured with alloantigen and rIL-12p70. nTreg were simulated with PVG stimulators and rIL-2 for 4 days to produce Ts1 cells that were washed and then re-stimulated for 3 days with PVG stimulators alone or with rIL-12p70, rIL-12p40, rIL-2, or control (supernatant from non-transfected CHO-K). Only rIL-2 (*p* < 0.05) and rIL-12p70 (*p* < 0.05) induced significant proliferation compared to controls, with no cytokine or with supernatant from non-transfected CHO-K cells. This induction of proliferation by rIL-12p70 demonstrated a functional significance of *il-12rβ2* expression by Ts1 cells.

Ts1 and Ts2 cells were generated by activation of nTreg with alloantigen and rIL-2 or rIL-4 respectively, as described (2). Percentage of cells expressing CD25 remained unchanged when cultured with alloantigen and either rIL-2 (Figure 2A) or rIL-4 (data not shown) (2), and 70–80% of cells expressed Foxp3 (2).

These Ts1 and Ts2 cells were first re-enriched for CD25^+^ cells to remove stimulator cells, before mRNA was extracted. Fresh nTreg did not express *il-12rβ2*, the specific receptor for IL-12p70. There was high expression of *il-12rβ2* on Ts1 cells (Figure 2B). Minimal expression of *il-12rβ2* was noted in nTreg cultured with autoantigen and rIL-2 and no expression with either auto- or alloantigen and rIL-4. There was no induction of *il-2* with any culture condition (results not shown), suggesting no Th1 cell induction. These results suggested that within 3–4 days of culture with rIL-2 and alloantigen, alloantigen-specific activated nTreg expressed *il-12rβ2*.

### rIL-12 p70 promoted proliferation of Ts1 cells

Re-culture of Ts1 with the same alloantigen for 4 days with rIL-2, rIL-12p70, or rIL-12p40 was performed (Figure 2C). With rIL-12p70 alone, proliferation was induced, though not as great as with rIL-2 alone. rIL-12p40 did not induce proliferation. Thus, rIL-12p70 acted as a growth factor for Ts1 cells albeit not as potent as rIL-2, a polyclonal activator of nTreg. The lesser response with rIL-12p70 is consistent with promotion of a small subpopulation of alloantigen-activated Treg rather than polyclonal activation of nTreg (2).

### rIL-12p70 enhanced regulatory activity of Ts1 cells

**Figure 3:**
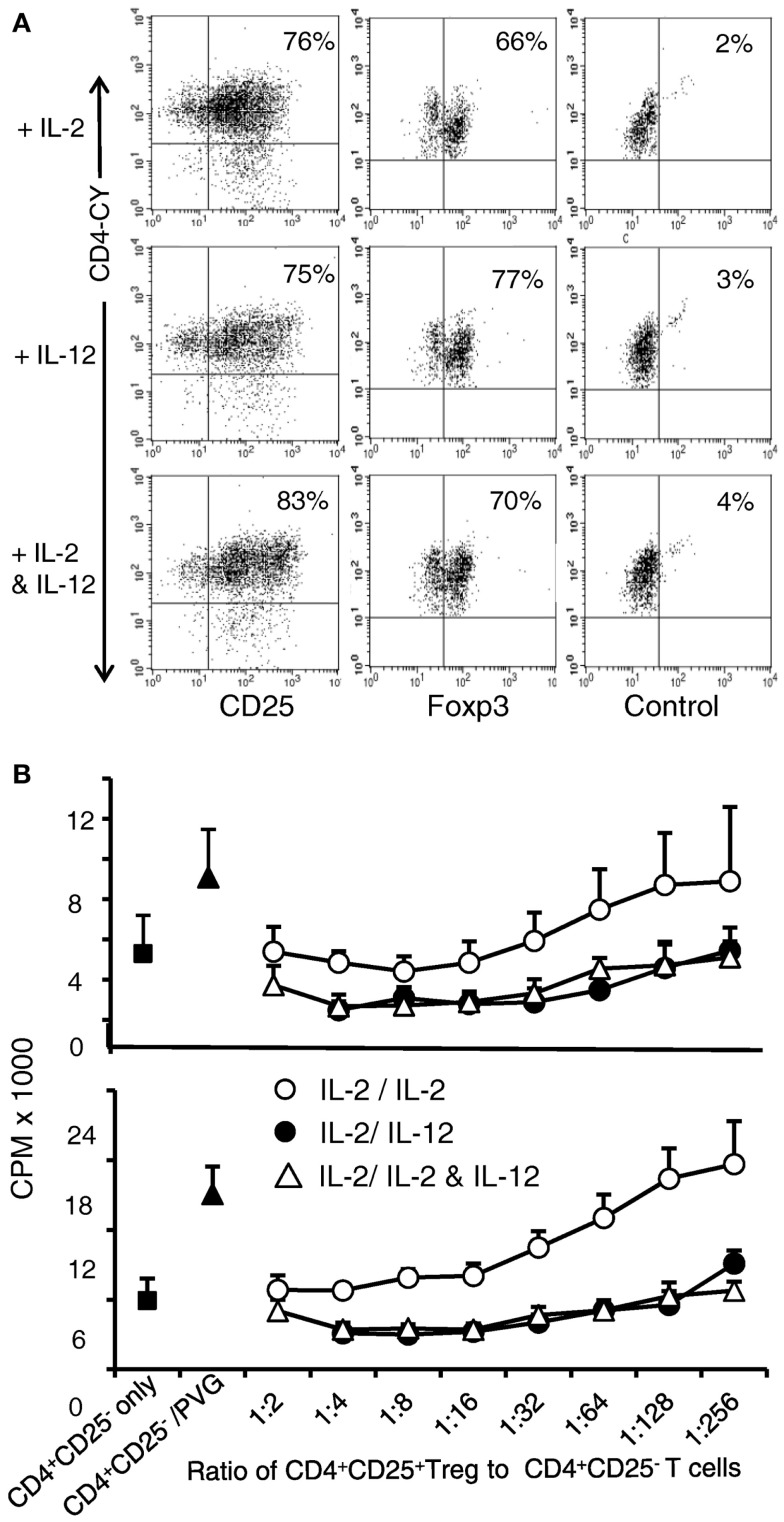
**Examination of phenotype and suppressive ability of Ts1 cells re-cultured with rIL-12p70 alone or in combination with IL-2**. Ts1 cells were washed and re-cultured with alloantigen and either rIL-2 alone, rIL-12p70 alone, or both rIL-2 and rIL-12p70 for 4 days before examination of: **(A)** CD25 and Foxp3 expression. All three cultures showed that a large proportion of cells were CD25^high^ and 70–80% expressed Foxp3. **(B)** Suppressive ability. Cells from all three cultures were tested for ability to suppress naive CD4^+^CD25^−^ T cell proliferation in MLC against PVG (top panel) or third party Lewis (lower panel) stimulator cells. A constant number (1 × 10^5^) of naïve CD4^+^CD25^−^ T cells were cultured with serial dilutions of re-cultured Ts1 cells. Ts1 re-cultured with PVG alloantigen and either rIL-12p70 (**●**) or rIL-2 and rIL-12p70 (**△**) had greater suppression compared to those re-stimulated with IL-2 alone (○). Proliferation was suppressed to or below background levels observed with naïve CD4^+^CD25^−^ T cells cultured alone (5.0 ± 1.2 × 10^3^ cpm) or with syngeneic DA stimulator cells (5.1 ± 2.0 cpm). Ts1 re-cultured with rIL-12p70 showed significant suppression at all dilutions (*p* < 0.001). Ts1 cells re-cultured with alloantigen and both rIL-2 and rIL-12p70 suppressed at dilutions to 1:256 (*p* < 0.0001). Ts1 cells re-cultured with rIL-2 and specific alloantigen, only suppressed to 1:32 (*p* < 0.001) showing no enhancement in suppressive ability of Ts1 cells that suppressed at 1:32–1:64 ratio to naive CD4^+^CD25^−^ T cells, as described (8). Suppression of CD4^+^CD25^−^ T cells response to third party Lewis alloantigen was similar to specific donor PVG. Thus, re-culture of Ts1 cells with specific alloantigen with rIL-12p70 increased their potency to suppress *in vitro*, whereas re-culture of Ts1 with rIL-2 and alloantigen did not. Similar results in two other experiments.

nTreg from DA rats were initially stimulated with rIL-2 and PVG alloantigen for 3 days to generate Ts1 cells, and were then re-cultured for 4 days with alloantigen and either rIL-2 alone, rIL-12p70 alone, or both rIL-2 and rIL-12p70. In all three cultures, a large proportion was CD25^+^ (75–83%) compared to >95% in the starting fresh naïve nTregs. Seventy to eighty percent of the cells continued to express Foxp3 (Figure 3A), similar to fresh naïve Treg. After 3 days of culture of nTreg with rIL-2 and alloantigen, the number of cells recovered was 34–43% of the starting number of nTreg. After re-culture of Ts1 cells for 4 days with rIL-2 alone or rIL-2 and rIL-12p70 the yields were 50–100% of the original cell number. After re-culture of Ts1 cells with rIL-12p70, the yield was 25–50% of the original Ts1 population. Culture with rIL-12p70 may select antigen-specific Treg, whereas rIL-2 polyclonally expands nTreg.

The suppressive ability of these re-cultured Ts1 on naïve CD4^+^CD25^−^ T proliferation to specific alloantigen was examined. This assay had serial dilutions of re-cultured Ts1, with the same number of stimulator and CD4^+^CD25^−^ T responder cells in all wells (Figure 3B). Without allogeneic stimulator cells, suppression was similar to background proliferation level of CD4^+^CD25^−^ T cells. Ts1 cells re-cultured with rIL-2 were unable to suppress at dilutions greater than 1:32–1:64 (2). In contrast, Ts1 cells cultured with rIL-12p70 alone or with rIL-2 and rIL-12p70 showed much enhanced suppressive ability and fully suppressed proliferation of CD4^+^CD25^−^ T cells to autologous background levels at 1:256. In other experiments, suppression of proliferation to background level was observed until 1:1024. This suppression was not antigen-specific, however (Figure 3B). In the methods of MLC that we use, naïve CD4^+^CD25^−^ T cells have a greater response to auto-antigen than CD4^+^ T cell as they lack nTreg. This proliferation to self is usually over 50% of that to alloantigen, as seen in this assay (9). Rat CD4^+^ T cells have lower levels of proliferation in MLC than humans and mice, thus these responses are normal for rat CD4^+^ T cells.

### Cytokine and cytokine receptor mRNA expression in Ts1 cells cultured with rIL-12p70, rIL-2, or rIL-2 and rIL-12p70

**Figure 4:**
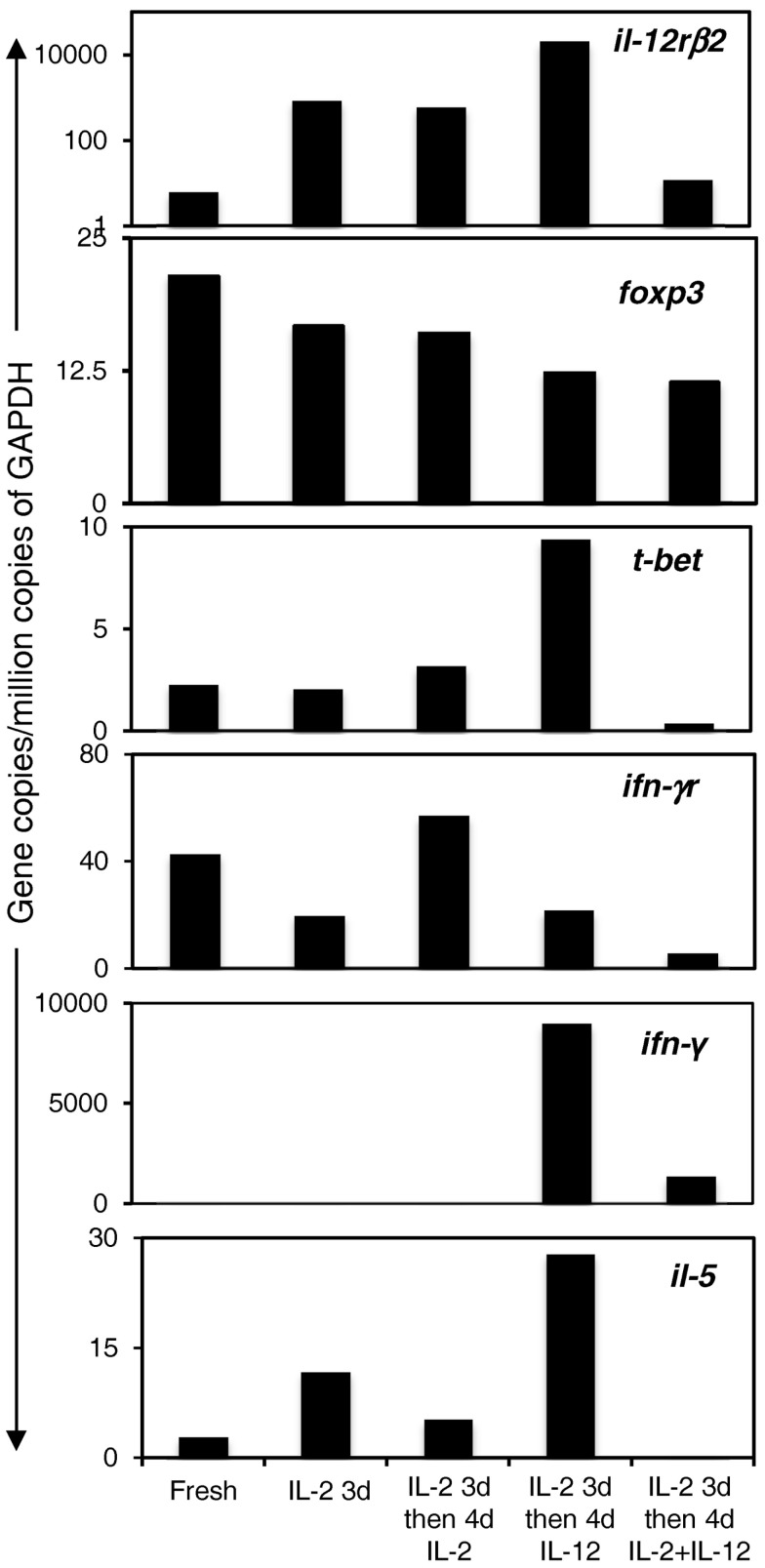
**Expression of transcription factor, cytokine, and cytokine receptor mRNA in Ts1 cells re-cultured with alloantigen and either rIL-2, rIL-12p70, or both rIL-2 and rIL-12p70**. Ts1 cells re-cultured with alloantigen and either rIL-12p70 alone or rIL-2 and rIL-12p70 had high expression of *foxp3* suggesting cells in all cultures were Treg. *il-12rβ2* levels were preserved in cultures with rIL-2 alone and rIL-12p70 alone but not those cultured with both rIL-2 and rIL-12p70. *ifngr* expression was enhanced with rIL-2, and was sustained with rIL-12p70 but not with both rIL-2 and rIL-12p70. *il-5* expression was increased with rIL-12p70 but waned with rIL-2 and was lost with rIL-2 and rIL-12p70. Induction of *t-bet*, the Th1 transcription factor, and *ifn-γ* occurred after re-culture with rIL-12p70 alone, and not after re-culture with rIL-2 or both rIL-2 and rIL-12p70. Thus, Ts1 cells re-cultured with rIL-12p70 alone developed a Th1-like Treg phenotype, while retaining *foxp3* expression and the CD4^+^CD25^+^Foxp3^+^ phenotype. Re-culture of Ts1 cells with rIL-2 retained the Ts1 phenotype. Re-culture of Ts1 cells with both rIL-2 and rIL-12p70 led to loss of the Ts1 phenotype and did not induce a Th1-like Treg phenotype.

Ts1 cells were re-cultured for 4 days with alloantigen and rIL-2 or rIL-12p70 or both rIL-2 and rIL-12p70 before enrichment with CD25-PE beads to eliminate stimulator cells. mRNA was extracted and subjected to RT-PCR for cytokine and cytokine receptor mRNA expression. We compared mRNA from these cells to mRNA from fresh nTreg and Ts1 cells (Figure 4). Expression of *il-12rβ2* was increased when Ts1 cells were re-cultured with rIL-2 alone or rIL-12p70 alone but markedly reduced when Ts1 cells were re-cultured with both rIL-2 and rIL-12p70. *Ifngr* expression was sustained by culture with IL-2, but was diminished in cultures with rIL-12p70.

FACS analysis showed Foxp3 expression was maintained after re-culture of Ts1 cells with rIL-12p70 alone (77% Foxp3^+^), rIL-2 alone (66% Foxp3^+^), and both rIL-2 and rIL-12p70 (70% Foxp3^+^).

*il-2* was not expressed in any culture conditions (data not shown), consistent with a Treg population, not a Th1 cell induction. The Th1 transcription factor *t-bet* and the Th1 cytokine *ifn-γ* were induced in Ts1 cells re-cultured with rIL-12p70 alone but not with rIL-2 alone or with both rIL-2 and rIL-12p70, consistent with a Th1-like Treg phenotype. The presence of rIL-2 in re-culture prevented induction of Th1-like Treg by rIL-12p70, as there was no induction of *t-bet* or *ifn-γ* mRNA.

Ts1 cells express *il-5* (2), and this expression was enhanced by culture with rIL-12p70 alone, but not in cultures with rIL-2.

Thus, re-culture of Ts1 cells with rIL-12p70 in the absence of rIL-2, induced a Th1-like Treg, which expressed more *il-12rβ2, t-bet, ifn-γ*, and *il-5* while retaining a Treg phenotype by expression of *foxp3* and no expression of *il-2*. *Ifngr* expression was less. They thus retained the Ts1 phenotype in that they expressed *foxp3, ifngr*, and *il-5* and were induced to express *t-bet* and *ifn-γ*. Re-culture of Ts1 with rIL-2 alone retained the Ts1 phenotype of *foxp3, ifngr, il-12rβ2*, and some *il-5*. Re-culture with both rIL-2 and rIL-12p70 led to loss of Ts1 phenotype and did not induce a Th1-like Treg phenotype, albeit they continued to express *foxp3* and did not express *il-2*.

### Examination of IFN-γ or iNOS as mediators of rIL-12p70 effect on Ts1

**Figure 5:**
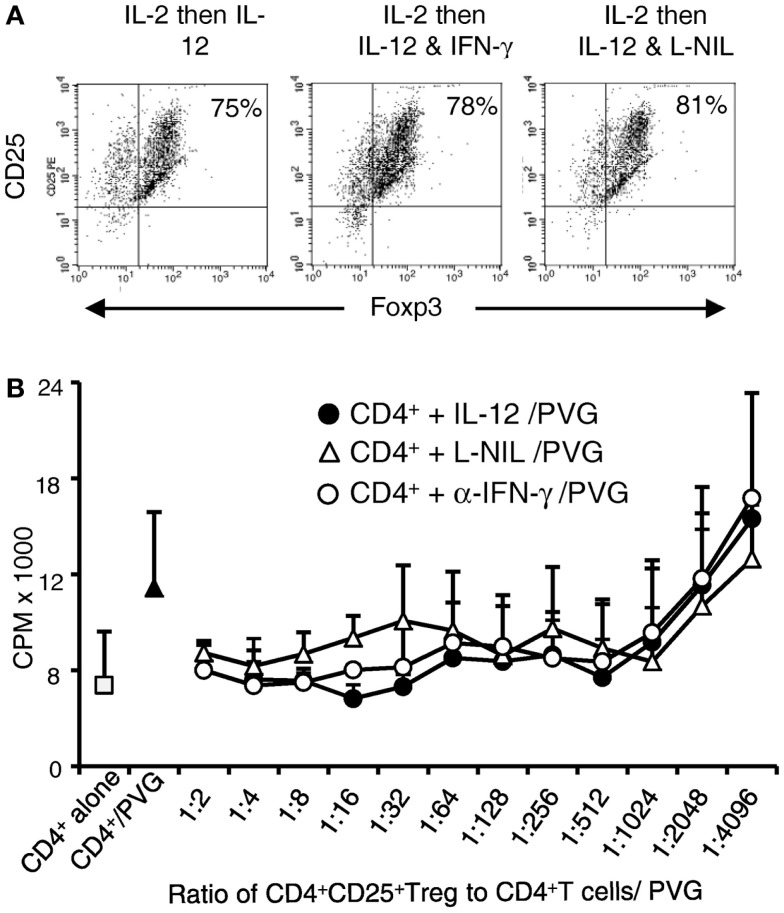
**Blocking IFN-γ or iNOS had no effect on alloantigen/IL-12p70 mediated enhancement of suppressive ability of Ts1 cells: Ts1 cells generated by culture of nTreg with PVG alloantigen and rIL-2 for 3 days were re-cultured with PVG alloantigen and either rIL-12p70 alone, rIL-12p70, and anti-IFN-γ (DB-1) or rIL-12p70 and L-NIL**. After culture **(A)** Foxp3 expression was maintained in all cultures with 75–81% expressing both CD25 and Foxp3. The starting nTreg and Ts1 populations had 70–80% Foxp3^+^ with nearly all were CD4^+^CD25^+^. **(B)** Blocking either IFN-γ or iNOS had no effect on the capacity of Ts1 cells re-cultured with rIL-12p70 and PVG stimulators to suppress naïve CD4^+^ T cell proliferation in MLC against specific PVG stimulators as measured by ^3^H-thymidine incorporation (cpm ×10^3^). MLC was significantly suppressed to or below background proliferation of control CD4^+^ T cells with no allogeneic stimulator (1.69 + 1.2 × 10^3^ cpm). Ts1 cells re-cultured with specific PVG stimulators and rIL-12p70 alone (●) had significantly suppressed proliferation of CD4^+^ T cells to PVG stimulators at all ratios of 1:4 through to 1:1024 (*p* < 0.02); for Ts1 cells re-cultured with rIL-12p70 and anti-IFN-γ (○), *p* < 0.015 to 1:512, and *p* = 0.02 at 1:1024. Ts1 re-cultured with rIL-12p70 and L-NIL (△) significant differences were from 1:4 through to 1:512; *p* < 0.004 except for 1:64 and 1:128. These results suggested rIL-12p70 directly acted on Ts1 cells, not via induction of IFN-γ and iNOS.

IL-12p70 prevents immune injury by induction of IFN-γ, which in turn induces iNOS leading to production of NO (29). Here, we found that re-culture of Ts1 with rIL-12p70 alone induced Th1-like Treg expressing *ifn-γ*. To examine if IFN-γ or iNOS played a role in the induction of Th1-like Treg, we compared Ts1 cells re-cultured with rIL-12p70 alone to those cultured with rIL-12p70 and either anti-IFN-γ antibody to block IFN-γ or L-NIL to inhibit iNOS. Post-culture, all three cell populations were >95% CD25^+^ and 75–81% Foxp3^+^ similar to the starting naïve nTreg and Ts1 cells (Figure 5A).

Cells from all three cultures were subjected to suppressor cell assay as in previous section for their ability to suppress proliferation of naïve DA CD4^+^ T cells to PVG (Figure 5B) and to Lewis alloantigen (results not shown). Proliferation of CD4^+^ T cells was suppressed with re-cultured Ts1 cells from all three cultures to levels similar to background proliferation. The proliferation was significantly less than the control response of naïve CD4^+^ T cells to PVG with no stimulators or no Treg added. These results suggested that rIL-12p70’s induction of Th1-like Treg was not through induction of IFN-γ or production of nitric oxide by iNOS.

### Capacity of IL-12p70 to induce Treg that suppress allograft rejection

**Figure 6:**
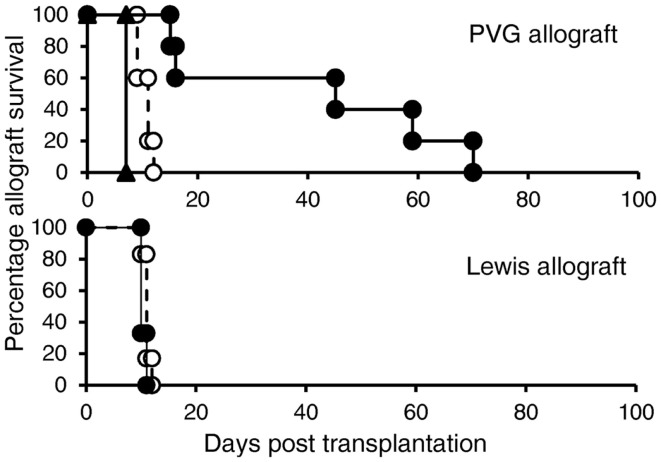
***In vivo* suppression of allograft rejection by Ts1 cells re-cultured with PVG stimulators and rIL-12p70**. Ts1 cells generated by culture of CD4^+^CD25^+^ T cells with PVG alloantigen and rIL-2 for 3 days were re-cultured with rIL-12p70 and PVG alloantigen for 4 days to produce Th1-like Treg (●). 5 × 10^6^ of the Th1-like Treg were given 3 days after grafting, as we expected by that time the rejection response producing IL-2, IL-12p70, and IFN-γ would be induced in the host, as described (32). These delayed PVG (top panel) but not third party Lewis (bottom panel) allograft rejection compared to normal rejection controls (○) (*n* = 5/group) (*p* < 0.01). In comparison, 5 × 10^6^ Ts1 cells that had been re-cultured with rIL-2 and PVG for 4 days (▴) did not delay rejection (*n* = 4), this demonstrated re-culture of Ts1 cells with rIL-12p70 was required to induce potent alloantigen-specific Treg that could suppress rejection.

The aim of this study was to induce potent alloantigen-specific Treg *in vitro* that alone could suppress allograft rejection *in vivo* without any other immunosuppression. Ts1 cells were re-cultured with rIL-12p70 and PVG alloantigen for 4 days to generate Th1-like Treg. 5 × 10^6^ of these Th1-like Treg cells were transferred to normal DA rats transplanted with PVG or Lewis heterotopic cardiac allografts without any immunosuppression. The Treg were given 3 days after transplant, at a time where the host will have activated T cells against the graft and there would be induction of IL-12. We reasoned that the Th1-like Treg would need IL-12 to survive and promote their further expansion. These Th1-like Treg significantly delayed PVG cardiac allograft rejection (12–70 days, median 46 days) compared to normal rejection of 7–10 days (*p* < 0.01) in this model. However, these Th1-like Treg activated against PVG alloantigen had no effect on third party Lewis graft rejection of 9–10 days, which was the same as control normal rejection time of 8–11 days (Figure 6). Ts1 cells re-cultured with rIL-2 and PVG alloantigen for another 4 days, did not delay rejection of PVG heterotopic cardiac allografts as all grafts were rejected in 7–8 days.

Adult rats have approximately 5 × 10^8^ peripheral CD4^+^ T cells, thus suppression by Th1-like Treg, occurred at a ratio of 1:100 of Treg to effector CD4^+^ T cells. 5 × 10^6^ activated CD4^+^CD25^+^ Treg given i.v. would not have markedly increased the peripheral CD4^+^CD25^+^ T cell pool, which is 3–5 × 10^7^ (4, 8, 9).

## Discussion

**Figure 7:**
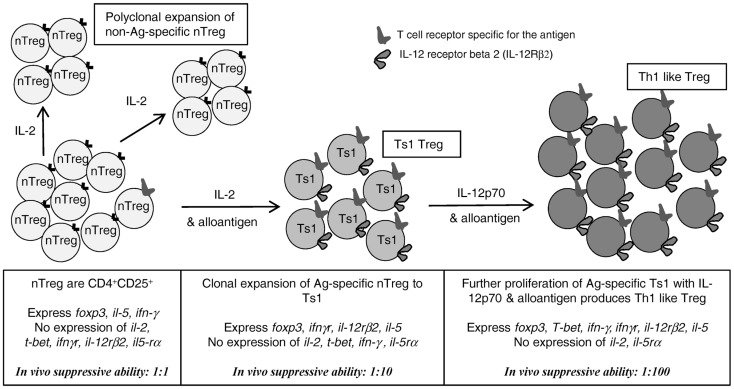
**Proposed pathway for activation/proliferation of alloantigen-specific nTreg and comparison with the non-antigen driven proliferation of nTreg that do not have a TCR specific for the antigen**. rIL-2, without antigen, induced proliferation of nTreg, which retain the characteristics of nTreg. In presence of antigen and rIL-2, nTreg with TCR for the stimulating antigen are first induced to express a Ts1 phenotype, including expression of *ifngr, il-12rβ2, foxp3*, and *il-5*. Stimulation of these Ts1 cells with alloantigen and rIL-12p70 in the absence of rIL-2, induced Th1-like Treg that retain the Ts1 phenotype, but in addition express *t-bet* and *ifn-γ* and no *il-2*. These have enhanced capacity to suppress *in vitro* and *in vivo* at lower ratios to effector CD4^+^ T cells, than nTreg (1:100 vs. 1:1) and Ts1 [1:100 vs. 1:10 (2)]. Thus, inflammatory Th1 cytokines drive induction of alloantigen-specific Treg; the first step is induction of alloantigen-specific Ts1 cells by the early Th1 cytokine IL-2; the second step is induced by rIL-12p70 only after IL-2 production wanes. rIL-12p70 induces Th1-like Treg that express both *t-bet* and *foxp3* as well as *ifn-γ, ifngr*, and *il-5*. Adapted from Figure 1 in Hall et al. (1).

These results support our hypothesis that activation of nTreg to alloantigen-specific Treg occurs in parallel with effector T cell activation (1, 2). With Th1 responses, initial activation is dependent on IL-2, the early Th1 cytokine. As IL-2 production wanes, the activated antigen-specific Treg appear to express *ifngr* and *il-12rβ2* suggesting Th1 cytokines IFN-γ and IL-12p70, whose expression persists after expression of IL-2 waned, may be required for survival of alloantigen-specific Treg (Figure 7). In this study, we identified that nTreg that had been activated by rIL-2 and alloantigen, that we describe as Ts1 (2), had their *in vitro* suppressive potency enhanced by further culture with rIL-12p70 and specific alloantigen. Indeed, Ts1 cells re-cultured with rIL-12p70 and specific alloantigen suppressed proliferation of naïve CD4^+^ T cells in MLC at a ratio of <1:1024. This suppression *in vitro* was not alloantigen-specific, however.

These Ts1 cells re-cultured with rIL-12p70 were potent antigen-specific suppressor cells *in vivo* that significantly delayed fully allogeneic specific donor but not third party donor heart graft rejection in normal hosts that received no other immunosuppressive treatment. DA rats can be readily induced to become tolerant to PVG (3, 35, 37, 39) and Lewis (36) organ allografts and the rate of induction of tolerance is similar. The same Th1-like Treg that suppressed PVG allograft rejection did not delay rejection of Lewis allograft, suggesting this was alloantigen-specific suppression. Thus, Th1-like Treg suppressed at a ratio of 1:100 or less to the host’s naïve CD4^+^CD25^−^ T cells.

Our studies used a heterogenous population of nTreg, that were 99% CD4^+^ and 98% CD25^+^ but only 70–80% Foxp3^+^. This is a standard preparation for murine studies. We used this population as we were examining practical ways of preparing antigen-specific Treg for therapy. The partial contribution of the CD4^+^CD25^+^Foxp3^−^population cannot be excluded, however there was no induction of *il-2*, suggesting Th1 cells were not activated.

These findings demonstrated that IL-12p70 is an important additional cytokine for the induction and full function of Treg of the Ts1 lineage, as has been suggested by others (21, 23) and reviewed in Hall et al. (1). Although proliferation and yield from cultures of Ts1 cells with rIL-12p70 alone was less than with rIL-2 alone the Th1-like Treg were more potent and thereby compensated for lower yield. As nTreg expressing TCR for specific antigen are a minor fraction of the whole nTreg population, any expansion of antigen-specific Treg will be associated with lower rates of proliferation and lower yields, than polyclonal activation with rIL-2.

We initiated these studies as the bioassay for IL-12p70 measures increased proliferation of IL-2 dependent T cells (38). We found rIL-12p70 but not rIL-12p40 promoted proliferation of rIL-2 alloactivated CD4^+^CD25^+^ nTreg. In previous studies, we described Ts1 cells express *ifngr*, a receptor for a late Th1 cytokine (2) and here we show that they also induced *il-12rβ2* expression. Ts2 cells generated by culture of nTreg with rIL-4 and alloantigen did not express *il-12rβ2* or *ifngr*, but expressed *il-5rα*, the receptor for the late Th2 cytokine IL-5 (2). rIL-12p70 in cultures of nTreg with rIL-2 and alloantigen enhanced their proliferation, but had no effect on nTreg in cultures with IL-4 and alloantigen. The Ts1 cells induced by alloantigen and rIL-2 proliferated when re-cultured with rIL-12p70 alone, demonstrating that IL-12Rβ2 was functional. In the presence of rIL-12p70, Ts1 cells were activated to Th1-like Treg expressing *T-bet, foxp3, ifn-γ, ifngr, il-12rβ2, il-5* but no *il-2*. This Th1-like Treg population displayed an enhanced ability to suppress the proliferation of naïve CD4^+^CD25^−^ T cells at a ratio of <1:1000 *in vitro* and <1:100 *in vivo*.

These findings extend our previous study that identified two separate lineages of Treg induced from nTreg by activation with alloantigen and either rIL-2 or rIL-4 (2). These strengthen our hypothesis that the initial activation of nTreg depends upon the early Th1 and Th2 cytokines, IL-2 or IL-4 to induce Ts1 and Ts2 cells, respectively (2). Within days, Ts1 cells become dependent for their growth and survival on other Th1 cytokines that are produced late in a Th1 response, as illustrated in Figure 7. In the case of Ts1, these appear to be IFN-γ and IL-12p70. This model proposes that development of nTreg into antigen-specific Treg is an inbuilt regulatory mechanism that is activated in parallel with Th1 responses (1). Further, this study identified a second step for the activation of potent alloantigen-specific Treg in presence of IL-12p70 alone that may occur when IL-2 production wanes. Thus, the induction of highly potent alloantigen-specific Treg during a Th1 response initially requires IL-2, but later may become dependent on IL-12p70 and possibly IFN-γ as reviewed in Ref. (1).

In our studies, it cannot be excluded that rIL-12p70’s effect is indirect, by activating a minority Th1 population in the cultures. This was manifest by the induction of *t-bet* and *ifn-γ* within the cells re-cultured with rIL-12p70 alone. It is possible that release of IFN-γ during a Th1 response activated by rIL-12p70, activated Ts1 cells by the IFNGR they express. An alternate possibility is that rIL-12p70 induced *t-bet* and *ifn-γ* in the Treg so they could produce IFN-γ to promote their own function and growth. Blocking anti-IFN-γ mAb did not prevent rIL-12p70 inducing highly suppressive Treg suggesting induction of Th1-like Treg did not depend on IFN-γ.

Many of the anti-inflammatory effects of IL-12p70 are attributed to production of IFN-γ that in turn induces iNOS to produce nitric oxide (29). Our studies show that any production of nitric oxide by iNOS is not essential for Th1-like Treg development *in vitro*. In other studies, we found the same concentration of L-NIL and anti-IFN-γ, enhanced proliferation of CD4^+^CD25^−^ T cells by blocking induction of iNOS and NO (9). Thus, in the current study L-NIL and IFN-γ would have been blocked, supporting the notion that IL-12p70 directly acts via the IL-12Rβ2 on Ts1 cells to induce proliferation and activation to more potent Th1-like Treg.

These findings are supported by recent reports of induction of Th1-like Treg during Th1 responses (23). These Th1-like Treg can be induced by IFN-γ or IL-12p70, and express *t-bet, stat1* and *ifn-γ*, as well as *foxp3*. Our studies demonstrated that the removal of rIL-2, and continued culture of Ts1 cells with rIL-12p70 induced a Th1-like Treg (Figure 7) (23). In man, Th1-like Treg have been described in patients with multiple sclerosis (26) and renal transplants (27).

IL-12p70 is a heterodimer composed of p35 and p40 and is a pro-inflammatory cytokine that enhances Th1 (40, 41), cytotoxic CD8^+^ T (42), and NK (43) cell responses by increasing IFN-γ production (44). IL-12p70 also promotes proliferation of IL-2 dependent T cells and enhances expression of CD25 on CD4^+^ Th1 cells (45) and clones (46) and on activated CD8^+^ T cells (42) IL-12p70 acts by binding to a high affinity receptor, which is a heterodimer of IL-12Rβ1 and IL-12Rβ2 (47). Resting T cells do not express high affinity *il-12rβ2* (48), but both chains are up-regulated by TCR and CD28 stimulation, as well as by IL-2 and IFN*-γ*. IL-4 and IL-10 decrease expression of *il-12rβ2*. Thus, *in vivo* where there are CD4^+^ and CD8^+^ T effectors, the effects of IL-12p70 may be more complex (49) than observed here with isolated CD4^+^CD25^+^ Treg. Our results suggest that CD4^+^CD25^+^Foxp3^+^ Treg activated by antigen and IL-2, like activated effector T cells, express il-12rβ2 and respond to IL-12p70.

Because IL-12p70 promotes induction of Th1 and cytotoxic T cell responses, it was predicted to play a key role in amplifying rejection and GVHD (50). Paradoxically, treatment with one dose of IL-12p70 at the time of bone marrow transfer inhibits fully allogeneic GVHD (51). Prevention of GVHD by rIL-12p70 is dependent on donor IFN-γ (52) and acts via Fas to inhibit donor T cell expansion (53). IL-12p70 treatment delays allograft rejection (29) and inhibits autoimmunity including uveitis (54) and EAE (22). The protective effects of IL-12p70 are associated with induction of *ifn-γ* and *inos* (54). Blocking IFN-γ or iNOS with L-NIL prevents IL-12p70 prolonging graft rejection (29). However, these studies did not examine any effect of IL-12p70 on Treg activation.

Further evidence that IL-12p70 can control autoimmune inflammation comes from enhanced autoimmunity in IL-12p35^−/−^ mice (55), IL-12Rβ2^−/−^ (56), IFN-γ^−/−^ (57), and IFNGR^−/−^ (58) mice compared to wild-type control mice.

Our study confirmed the role of IL-12p70 in induction of Th1-like Treg (21, 23) and demonstrated another mechanism by which IL-12p70 may down-regulate immune responses.

Current preclinical and early clinical studies use nTreg that have been expanded in numbers by repeated culture of nTreg with IL-2 and mAb to CD3 and in some studies to CD28. However, these cells remain nTreg phenotype and are required at a physiological ratio of >1:4, and usually 1:1 to effector cells to suppress GVHD (11, 14) and allograft rejection (8).

Our study identifies a way to expand, within a week, highly potent alloantigen-specific Treg that can suppress allograft rejection. We hypothesize that nTreg with TCR for the specific alloantigen express *ifngr* (2) and *il-12rβ2* (Figure 7) and have increased potency of suppression (2). At the same time, there is IL-2 induced polyclonal expansion of nTreg, which remain nTreg and are not induced to express *ifngr* and *il-12rβ2*, as illustrated in Figure 7.

To our knowledge, this is the first description of a Treg with antigen-specificity that can delay fully allogeneic graft rejection in unmodified recipients. The relatively short culture period required to induce Th1-like Treg and the small number required to suppress fully allogeneic graft rejection provide potential for generation of Th1-like Treg *in vitro* for clinical use.

## Author Contributions

Bruce Milne Hall, Nirupama Darshan Verma, Suzanne J. Hodgkinson, Karren Michelle Plain, and Giang T. Tran initiated and designed the research, Nirupama Darshan Verma, Karren Michelle Plain, Giang T. Tran, Catherine M. Robinson, Rochelle Boyd performed the research. Chuanmin Wang, Rochelle Boyd, and G. Alex Bishop did the cardiac transplants, Nirupama Darshan Verma, Bruce Milne Hall, Suzanne J. Hodgkinson, Giang T. Tran, Catherine M. Robinson analyzed the data, Bruce Milne Hall, Nirupama Darshan Verma, G. Alex Bishop, Suzanne J. Hodgkinson, and Giang T. Tran wrote the paper.

## Conflict of Interest Statement

Bruce Milne Hall and Suzanne J. Hodgkinson hold patents or pending patents related to cytokine activation of antigen activated Treg. All other authors have no competing financial interests.
